# Supplementary material for: Heterozygous and homozygous variants in STX1A cause a neurodevelopmental disorder with or without epilepsy
Source: Eur J Hum Genet. 2022 Dec 23;31(3):345–52. doi: 10.1038/s41431-022-01269-6 (PMC9995539; doi:10.1038/s41431-022-01269-6)
Supplement: Supplementary file 1 — Supplementary Material [file 41431_2022_1269_MOESM1_ESM.pdf]

Heterozygous and homozygous variants in *STX1A* cause a neurodevelopmental disorder with or without epilepsy

Johannes Luppe, Heinrich Sticht, François Lecoquierre, Alice Goldenberg, Kathleen Gorman, Ben Molloy, Emanuele Agolini, Antonio Novelli , Silvana Briuglia, Outi Kuismin, Carlo Marcelis, Antonio Vitobello, Anne-Sophie Denommé-Pichon, Sophie Julia, Johannes R. Lemke, Rami Abou Jamra, Konrad Platzer

Content

Figure S1. Pedigree of the family of Individuals 1 and 2. 2
Table S1. Detailed phenotypic overview of all individuals with rare variants in STX1A 3
Table S2. *In silico* prediction of splice variant in *STX1A* 4
Table S3. *In silico* prediction of missense and inframe deletion variants in *STX1A* 5
Table S4. Variant classification according to ACMG criteria 6
References.... 7

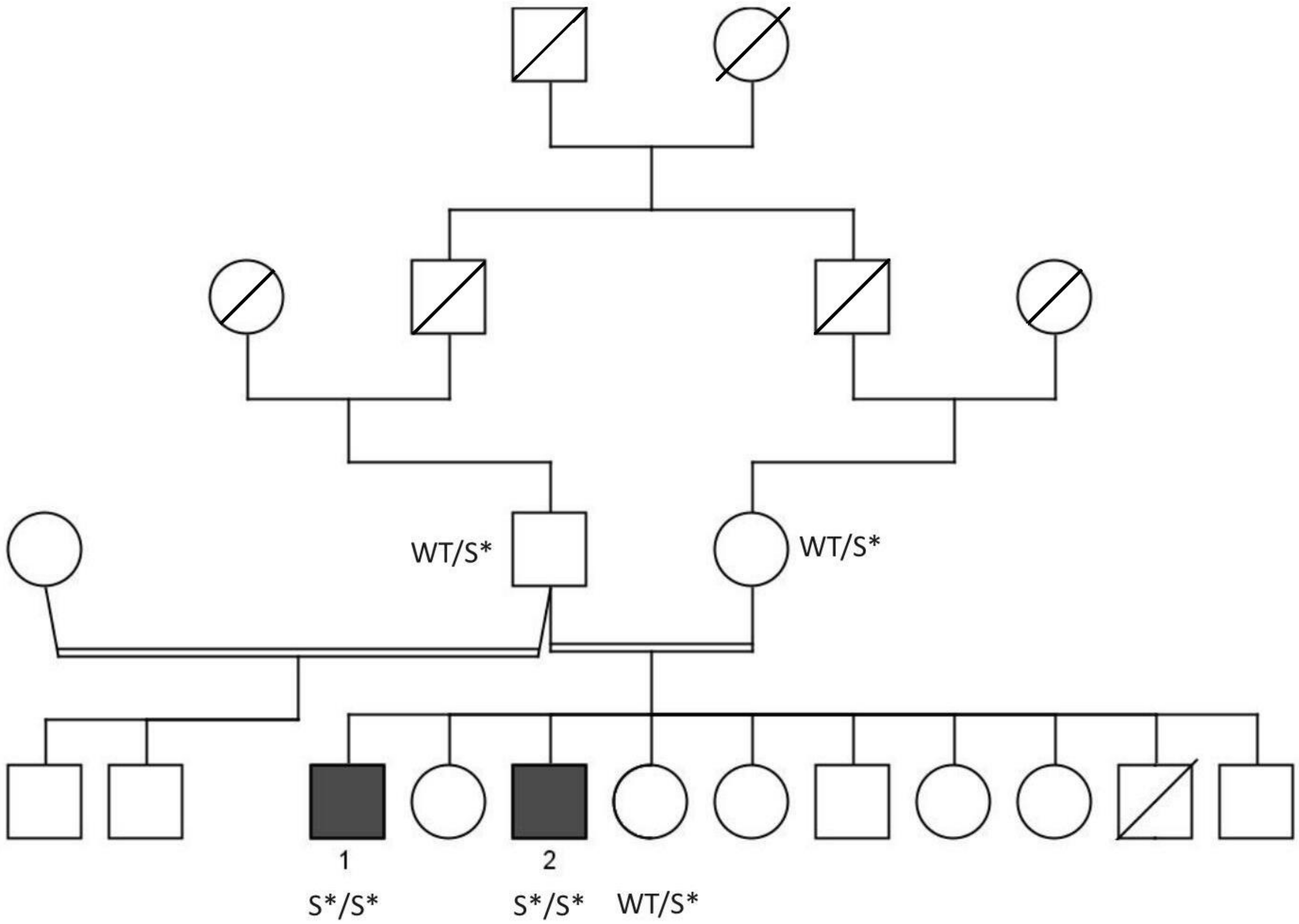

Figure S1. Pedigree of the family of Individuals 1 and 2.

Affected individuals 1 and 2 are both displayed as filled out black squares and were both homozygous for the splice variant *STX1A*:c.284-1G>A, p.?. Both parents and the sister born in between the two affected individuals were tested to be heterozygous for this variant. Another deceased brother was affected by an epidermolysis bullosa like phenotype (square with diagonal line in the latest generation). Genotypes of tested family members are shown next the respective individual. Abbreviations: WT: wildtype STX1A, S* splice variant *STX1A*:c.284-1G>A p.?

Table S1. Detailed phenotypic overview of all individuals with rare variants in STX1A

Provided as a separate Excel-file.

Table S2. *In silico* prediction of splice variant in *STX1A*

Ind.	Genomic position (hg19)	variant, NM_004603.4	CADD-v6[1]	SpliceAI[2]	MaxEntScan[3]	NNSPLICE[4]	Nucleotide conservation	gnomAD[5]
1, 2	chr7:73118754	c.284-1G>A, p.?	35	0.95	1	1	high	0

Color code represents the probability of the variant to be damaging; red: high. The family of individuals 1 and 2 of Syrian descent were lost to follow-up to perform RNA analysis of the proposed splicing defect.

Table S3. *In silico* prediction of missense and inframe deletion variants in *STX1A*

Ind.	Genomic position (hg19)	Variant NM_004603.4	CADD-v6[1]	REVEL[6]	Mutation Taster[7]	M-CAP 1.3[8]	Polyphen 2 v2.2.2[9]	AA conservation	gnomAD[5]
3, 4	chr7:73118509	c.435C>G, p.(Cys145Trp)	29.2	0.547 (LDC)	D	0.109 (PoP)	PrD	high	0
5	chr7:73117299	c.554C>G, p.(Ser185Cys)	24.9	0.301 (MDC)	D	0.032 (PoP)	B	high	0
6	chr7:73117183_73117185del	c.668_670del, p.(Val223del)	22.1	NA	NA	NA	NA	high	0
7	chr7:73117176	c.677A>G, p.(Gln226Arg)	32	0.723 (LDC)	D	0.328 (PoP)	PrD	high	0
8	chr7:73115125_73115127del	c.722_724del, p.(Val241del)	20.4	NA	NA	NA	NA	high	0
(55498)[10]	chr7:73119527	c.236T>G, p.(Met79Arg)	23.8	0.238 (MDC)	D	0.021 (LB)	B	high	0

Color code represents the probability of the variant to be damaging; red: high; orange: medium; green: low; LDC = likely disease causing; LDC = maybe disease causing; D = Deleterious; PoP = Possibly Pathogenic; PrD = Probably Damaging; B = Benign; LB = likely benign; NA = not available

Table S4. Variant classification according to ACMG criteria

Ind.	Genomic position (hg19)	Variant NM_004603.	Allelic state	ACMG criteria[11,*]	Classification
1, 2	chr7:73118754	c.284-1G>A, p.?	Homozygous	PVS1, PM2_SUP, PS4_SUP	Pathogenic
3, 4	chr7:73118509	c.435C>G, p.(Cys145Trp)	heterozygous, de novo	PS2, PM2_SUP, PS4_SUP, PP2, PP3	Likely pathogenic
5	chr7:73117299	c.554C>G, p.(Ser185Cys)	heterozygous, de novo	PS2, PM2_SUP, PP2, PP3	Likely pathogenic
6	chr7:73117183_73117185del	c.668_670del, p.(Val223del)	heterozygous	PM4, PM2_SUP, PP2, PP3	Uncertain Significance**
7	chr7:73117176	c.677A>G, p.(Gln226Arg)	heterozygous, de novo	PS2, PM2_SUP, PP2, PP3	Likely pathogenic
8	chr7:73115125_73115127del	c.722_724del, p.(Val241del)	heterozygous, de novo	PS2, PM4, PM2_SUP, PP2, PP3	Likely pathogenic
(55498)[10]	chr7:73119527	c.236T>G, p.(Met79Arg)	heterozygous, de novo	PS2_SUP, PM2_SUP, PP2, PP3	Uncertain Significance

* Although ACMG criteria are formally not suited for the description of novel disease-associated genes, we classified all variants from this study to simplify further use of the data.

** Due to the clinical overlap to the rest of the cohort, this variant is deemed causative despite being classified as uncertain (due to the lack of parental testing on de novo status).

References

1. Rentzsch P, Schubach M, Shendure J, Kircher M. CADD-Splice—improving genome-wide variant effect prediction using deep learning-derived splice scores. *Genome Med*. 2021;13(1):31. doi:10.1186/s13073-021-00835-9

2. Jaganathan K, Kyriazopoulou Panagiotopoulou S, McRae JF, et al. Predicting Splicing from Primary Sequence with Deep Learning. *Cell*. 2019;176(3):535-548.e24. doi:10.1016/j.cell.2018.12.015

3. Yeo G, Burge CB. Maximum entropy modeling of short sequence motifs with applications to RNA splicing signals. *J Comput Biol*. 2004;11(2-3):377-394. doi:10.1089/1066527041410418

4. Jian X, Boerwinkle E, Liu X. In silico prediction of splice-altering single nucleotide variants in the human genome. *Nucleic Acids Res*. 2014;42(22):13534-13544. doi:10.1093/nar/gku1206

5. Karczewski KJ, Francioli LC, Tiao G, et al. The mutational constraint spectrum quantified from variation in 141,456 humans. *Nature*. 2020;581(7809):434-443. doi:10.1038/s41586-020-2308-7

6. Ioannidis NM, Rothstein JH, Pejaver V, et al. REVEL: An Ensemble Method for Predicting the Pathogenicity of Rare Missense Variants. *The American Journal of Human Genetics*. 2016;99(4):877-885. doi:10.1016/j.ajhg.2016.08.016

7. Schwarz JM, Rödelsperger C, Schuelke M, Seelow D. MutationTaster evaluates disease-causing potential of sequence alterations. *Nat Methods*. 2010;7(8):575-576. doi:10.1038/nmeth0810-575

8. Jagadeesh KA, Wenger AM, Berger MJ, et al. M-CAP eliminates a majority of variants of uncertain significance in clinical exomes at high sensitivity. *Nat Genet*. 2016;48(12):1581-1586. doi:10.1038/ng.3703

9. Adzhubei I, Jordan DM, Sunyaev SR. Predicting functional effect of human missense mutations using PolyPhen-2. *Curr Protoc Hum Genet*. 2013;Chapter 7:Unit7.20. doi:10.1002/0471142905.hg0720s76

10. Kaplanis J, Samocha KE, Wiel L, et al. Evidence for 28 genetic disorders discovered by combining healthcare and research data. *Nature*. 2020;586(7831):757-762. doi:10.1038/s41586-020-2832-5

11. Richards S, Aziz N, Bale S, et al. Standards and guidelines for the interpretation of sequence variants: a joint consensus recommendation of the American College of Medical Genetics and Genomics and the Association for Molecular Pathology. *Genetics in Medicine*. 2015;17(5):405-424. doi:10.1038/gim.2015.30
